# Towards the development of a DNA-sequence based approach to serotyping of *Salmonella enterica*

**DOI:** 10.1186/1471-2180-4-31

**Published:** 2004-08-06

**Authors:** Chloe KB Mortimer, Tansy M Peters, Saheer E Gharbia, Julie MJ Logan, Catherine Arnold

**Affiliations:** 1 Special and Reference Microbiology Division, Health Protection Agency, Colindale, London, UK

## Abstract

**Background:**

The *fliC *and *fljB *genes in *Salmonella *code for the phase 1 (H1) and phase 2 (H2) flagellin respectively, the *rfb *cluster encodes the majority of enzymes for polysaccharide (O) antigen biosynthesis, together they determine the antigenic profile by which *Salmonella *are identified. Sequencing and characterisation of *fliC *was performed in the development of a molecular serotyping technique.

**Results:**

*FliC *sequencing of 106 strains revealed two groups; the g-complex included those exhibiting "g" or "m,t" antigenic factors, and the non-g strains which formed a second more diverse group. Variation in *fliC *was characterised and sero-specific motifs identified. Furthermore, it was possible to identify differences in certain H antigens that are not detected by traditional serotyping. A rapid short sequencing assay was developed to target serotype-specific sequence motifs in *fliC*. The assay was evaluated for identification of H1 antigens with a panel of 55 strains.

**Conclusion:**

*FliC *sequences were obtained for more than 100 strains comprising 29 different H1 alleles. Unique pyrosequencing profiles corresponding to the H1 component of the serotype were generated reproducibly for the 23 alleles represented in the evaluation panel. Short read sequence assays can now be used to identify *fliC *alleles in approximately 97% of the 50 medically most important *Salmonella *in England and Wales. Capability for high throughput testing and automation give these assays considerable advantages over traditional methods.

## Background

*Salmonella *express flagellar (H), polysaccharide (O) and capsular (Vi) antigens which determine strain pathogenicity and therefore variation of these antigens has formed the basis for *Salmonella *serotyping. The Kauffmann-White scheme, first published in 1929, divides *Salmonella *into more than 2500 serotypes according to their antigenic formulae. Within these, 46 O antigen groups are recognised by *Salmonella *serotyping. O antigen synthesis and assembly is encoded by the *rfb *gene cluster which typically contains 12 open reading frames, and ranges in size between serotypes, from approximately 8 kbp to 23 kbp. The variation of O antigens is not due to individual gene sequence variation, but rather to different sets of genes [1]. Approximately 20,000 repeating flagellin proteins polymerise to form the flagellar filament. The ends of the protein are conserved and responsible for the hairpin shape of the subunit while variation in the central region generates the antigenic diversity. Most serotypes exhibit diphasic flagellar antigen expression by alternately expressing two genes, *fliC *(phase 1) and *fljB *(phase 2) which encode flagellins of different antigenicity. *Salmonella *serotyping methods recognise 63 distinct phase 1 flagellar antigenic factors and 37 phase 2 flagellar antigenic factors although the latter are not always present. Some antigenic factors, denoted by square brackets in formulae, may be present or absent without affecting serotype designation. Serotyping methods are stable, reproducible and have high typeability, yet there are several drawbacks, particularly the dependence on availability of antisera considering the ethics, cost and quality control measures necessary to maintain such a supply.

Pulsed-field gel electrophoresis (PFGE) [2,3] is currently the bench-mark for molecular subtyping of *Salmonella*, however it is best used in combination with plasmid profiling and ribotyping for strain discrimination for epidemiological purposes [4]. Other approaches include fluorescent amplified fragment length polymorphism (FAFLP) [5] and multi-locus enzyme electrophoresis (MLEE) [6] which sample genomic DNA and provide a view of genetic diversity between strains and partially group some serotypes, but on the whole do not group or identify serotypes. Multi-locus sequence typing (MLST) has been used to discriminate between *Salmonella *strains by sampling variation in a set of housekeeping genes which precludes antigen encoding genes [7]. In 1993, Luk *et al *[8] published a length heterogeneity PCR (LH-PCR)-based method that targeted genes only associated with particular O antigens (A, B, C2 and D), while a more recent study by Fitzgerald *et al *[9] developed a serotype specific PCR assay targeting a single O serotype (O:6,14). Several studies have used a molecular approach to discriminate between particular flagellar serotypes (9, 11–12). *FliC *fragment restriction patterns using a dual enzyme combination allowed differentiation of flagellar types b, i, d, j, l,v, and z_10 _but r and e,h nor [f],g,m, [p], g,p, and g,m,s could be separated using this technique [10]. Hong used restriction fragment patterns of *fliC *and *fljB *for serotyping of poultry *Salmonella *but could not distinguish *S*. Enteritidis from *S*. Gallinarum and *S*. Dublin [11]. Design of a multiplex-polymerase chain reaction (multiplex-PCR) to identify 1,2, 1,5, 1,6, 1,7, 1,w, e,n,x and e,n,z_15 _second-phase antigens has been reported [12]. Peters and Threlfall reported *fliC *restriction fragment length polymorphism (RFLP) profiles were not specific enough to differentiate between certain serotypes [13]. To date no studies have attempted a universal molecular serotyping approach. Relevant publicly available sequence data is incomplete, as is epitope mapping information about specific serotypes, therefore approaches are currently being explored to characterise the expressed antigen or the encoding genes as an alternative to traditional serotyping.

For *fliC*, evidence from antibody binding studies suggests that sequences of ~300 nucleotides of the central variable region of flagellin correlate with serotype [14] and differences in amino acid sequence can be associated with differences in antigenic specificity. Comparative sequencing has distinguished some *salmonella* serotypes or biotypes [15-18]. Previous studies have provided full gene sequence for 19 phase 1 flagellar types. The need for a robust single molecular technology to discriminate different serotypes is clear, however sequence data representing all 63 recognised phase 1 flagellar types is incomplete. The aim of this study was to generate full gene sequences for representatives of the majority of phase 1 flagellar serotypes with a view to identifying serotype-specific motifs. These were then used to design a short sequence- or single nucleotide polymorphism-(SNP) based assay targeting characteristic motifs using pyrosequencing. This technology is based on sequencing by synthesis; four nucleotides are added step-wise to a primer-template mix. Incorporation of a nucleotide i.e. extension of the DNA strand, leads to an enzymatic reaction resulting in a light flash. A pyrogram is produced from which the template DNA sequence is deduced. The assay was validated on a panel of 55 strains to initiate a DNA sequence based approach for serotyping *Salmonella enterica*.

## Results and discussion

**Figure 1:**
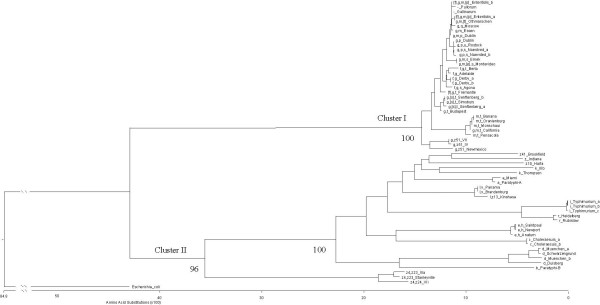
Protein distance tree of H1 antigens. The tree displays the inferred amino acid sequence distances between full H1 antigens. The label displays the H1 antigen, and the *Salmonella *serotype or subgroup from which the sequence was obtained. *Esherichia coli fliC*-*H7 *sequence was used to root the tree. Bootstrap values are displayed at major nodes. Sequences labelled with _a, _b or _c indicate an H1 allele found to be encoded by multiple sequences (Additional file 2).

Alignment of 106 *fliC *sequences generated in this study and 32 phase 1 flagellin sequences previously published (see Methods section), representing 35 phase 1 flagellar serotypes revealed a clear division of sequences into two groups. Representative sequences are aligned in Additional file 1. A tree indicating the relatedness of these sequences generated from translated DNA sequence supported this division with a 100% bootstrap value (Figure 1). Sequences encoding phase 1 flagellar antigens exhibiting antigenic factors "g" or "m,t" are referred to as members of the g-complex and the *fliC *sequences of this group clustered exclusively with the non-motile strains Gallinarum and Pullorum on the tree (Cluster I, Figure 1). The level of amino acid sequence homology within Cluster I sequences was 90.05%. Sequences not encoding the antigenic factors "g" or "m,t", formed the second group of sequences (Cluster II), referred to here as the non-g complex. Lower levels (80.3 %) of amino acid similarity were observed within Cluster II. Sero-specific polymorphisms were identified within the central variable region where consensus sequences of Cluster I and Cluster II diverged, between amino acid positions 160 – 407 (based on amino acid numbering system of the sequenced strain of *S*. Typhimurium (AE008787) represented here as sequence type Typhimurium_a).

**Figure 3:**
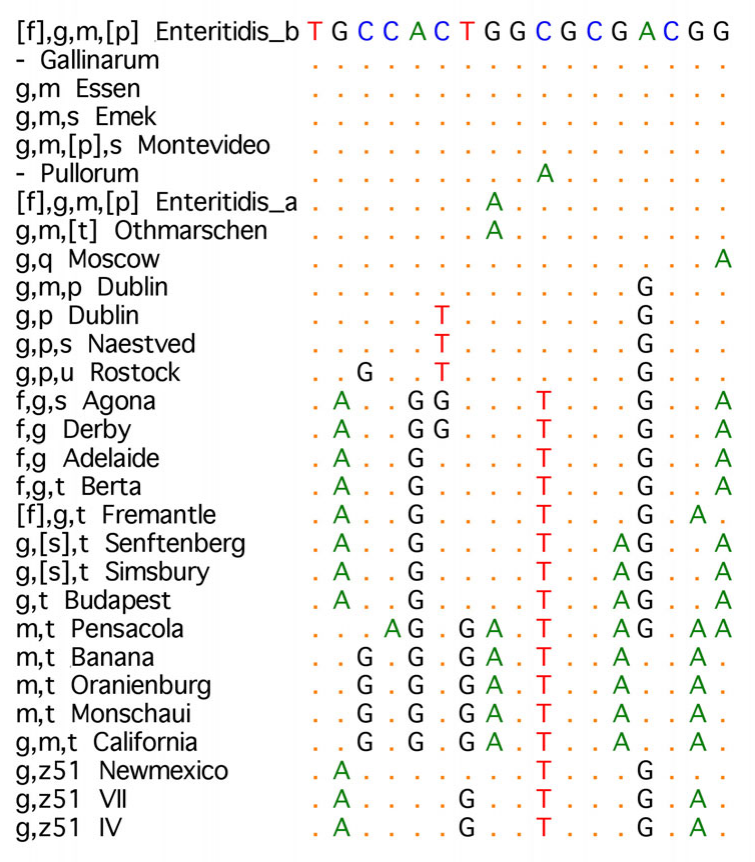
Sequence motifs at target g. The assay for g-complex strains detected 17 bp of sequence commencing at nucleotide position 917. Fifteen sequence types were identified and differentiated between H1 serotypes.

**Figure 4:**
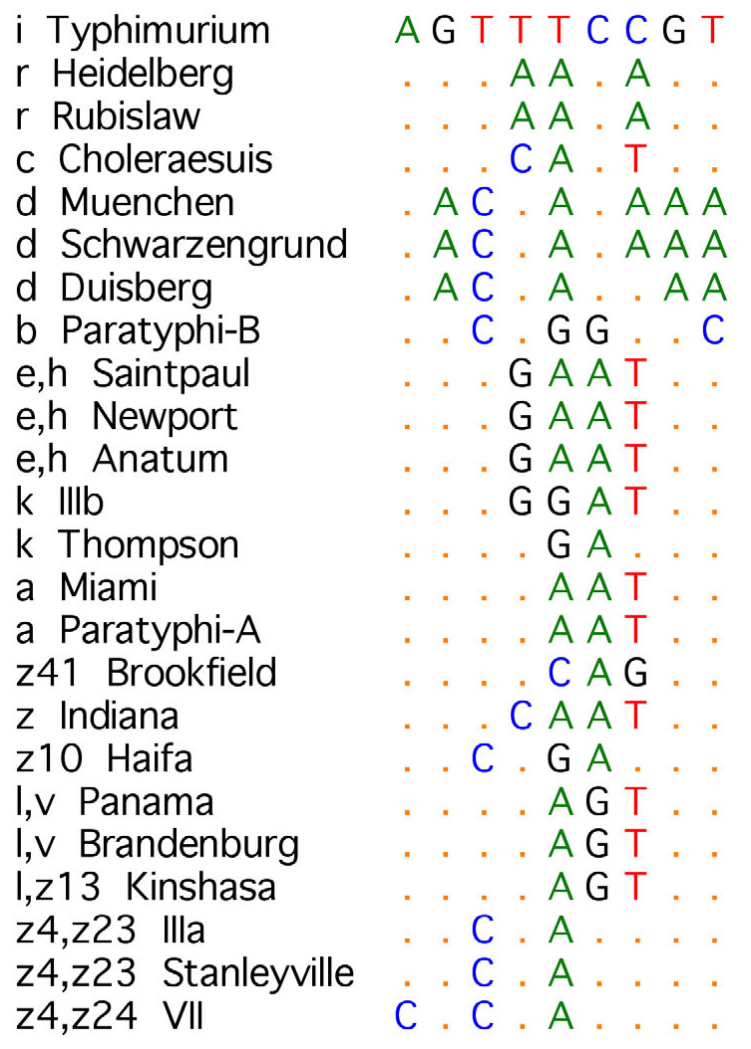
Sequence motifs at target non-g. The assay for non-g complex strains detected 9 bp of sequence commencing at nucleotide position 739. Sixteen sequence types were identified among non-g complex strains tested.

**Figure 5:**
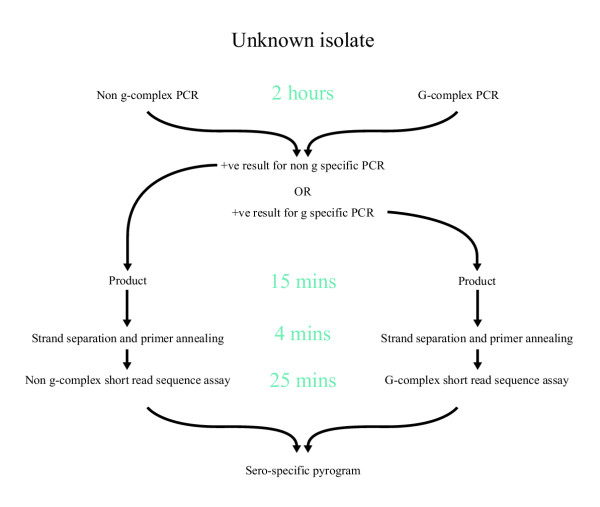
Algorithm for identification of unknown isolates. Times given are approximate for 96 samples using methods described.

*Salmonella fliC *sequences were conserved at their termini and variable in the central region between serotypes [16,18] and clustered according to allele. Amino acid and nucleotide positions described here-in are with reference to the sequenced strain LT2. It was apparent from the alignment of sequences generated in this study that two assays were required, one encompassing Cluster I strains and one for Cluster II. Multiple alignments were created for each cluster and regions of the *fliC *gene containing sero-specific polymorphisms were identified at nucleotide positions 917 – 933 and 739–749 in Cluster I and Cluster II respectively (Figures 3 and 4). PCR primers were designed to amplify the target region in each sequence (see below). One multiplex PCR was developed for each group containing a mixture of specific primers. All primers designed for short sequence assays in this study are shown in Additional file 3 and the testing algorithm is shown in Figure 5.

### Summary of *fliC *sequence variation within the g-complex

**Figure 2:**
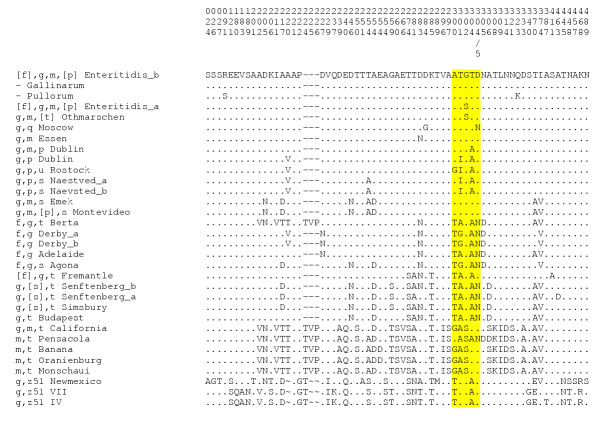
Amino acid polymorphisms among *fliC *of g-complex strains. Alignment displaying polymorphic codons only of g-complex *fliC *genes. Codon numbering is based on LT2 sequence, a slash is used where codons fall between LT2 codons in alignment. Highlighted area indicates region analysed in g-complex assay.

All polymorphisms within the g-complex sequences analysed are displayed in Figure 2 The target region (highlighted) was selected because it conferred multiple sero-specific amino acid substitutions and was variable at the DNA level. In the 17 bp nucleotide sequence assayed, 15 sequence types were identified (Figure 3). This region was assayed against the test panel of 17 *Salmonella *strains belonging to the g-complex and was able to exclusively identify sequence motifs corresponding to phase 1 flagellar serotypes. The serotypes not differentiated by this assay ([f],g,m, [p], g,m, g,m,s and g,m, [p],s or non-motile Gallinarum) were known from full sequencing to be identical at the target region.

### Amino acid differences between g-complex strains identified by full sequencing

The following polymorphisms located in *fliC *of the g-complex are likely to be involved in specific epitope formation: two amino acid sequence types were observed in 25 *fliC-[f],g,m, [p] *sequences obtained from *Salmonella enterica *serovar Enteritidis strains. Twenty-three *S*. Enteritidis strains demonstrated complete conservation in their DNA sequence (B16, B18, JTCM02 and 20 phage type 4 strains (Enteritidis_b)). The sequence of B17 was congruent with published *S*. Enteritidis (M84980) (Enteritidis_a), and exhibited a single amino acid (Ser>Gly at 302) substitution compared to sequence type Enteritidis_b. Published *S*. Othmarschen (U06455) *fliC-g,m*, *[t] *sequence inferred the same amino acid sequence as Enteritidis_a but exhibited a silent mutation at the DNA level. As the *fliC *sequence for these two serotypes was identical it was apparent that the sequence included here represented an *S*. Othmarschen strain in which the t factor was absent.

Published *S*. Gallinarum sequences demonstrated 100% DNA homology to Enteritidis_b except for a SNP encoding a stop codon in M84975. *S*. Pullorum and *S*. Gallinarum are non-motile as they do not express flagella. Antisera to the g factor antigen react strongly with induced-motility *S*. Pullorum culture, indicating that g epitopes are expressed in these cells [19]. This correlates with our sequence data as S. Pullorum clusters with g,m sequences (Figure 1). Biotype-specific polymorphisms for *S*. Pullorum were observed at amino acid position 91 and 323. Molecular identification of *S*. Pullorum and *S*. Gallinarum would be of considerable benefit as standard serotyping cannot differentiate these two serotypes.

*FliC-g,q *was differentiated from all other g-complex sequences by an Asp>Gly serotype-specific polymorphism observed at position 284 for *S*. Moscow. A Thr>Ala substitution at residue 304 conferred by a single nucleotide polymorphism (SNP) was identified between sequences of g,m and g,p, congruent with a previous report [20], and forms the basis for differentiation of these two serotypes. DNA polymorphisms, but no inferred amino acid substitutions, were observed between strains exhibiting g,m,s and g,m, [p],s. The p factor was not coded for by the *fliC *sequences of these strains.

*S*. Essen *fliC-g,m *was distinct from other g and m coding sequences by an Asp>Asn substitution at 283. *fliC-g,p,s *could be differentiated from *fliC-g,p *by a Thr>Ala substitution at 254. A motif of two amino acids at positions 302 and 307 was common to *S*. Derby, *S*. Agona, *S*. Adelaide, and *S*. Berta which exhibit phase 1 flagellar antigenic factors "f" and "g". This motif was exclusive to these serotypes. DNA sequence variation at corresponding positions allowed *S*. Derby and *S*. Agona to be distinguished from *S*. Adelaide and *S*. Berta. *FliC-g,z*_51_; and *fliC-m,t *with *fliC-g,m,t *each form distinct clusters (Figure 1).

### Summary of *fliC *sequence variation within the non-g complex

Sequence conservation within alleles that did not encode g or m,t antigenic factors was demonstrated by 97.8 – 99.1% homology and 80.35% homology was measured in the complex. The high level of variability between alleles in this group did not allow association of specific amino acids to epitope formation that was possible with the g-complex sequences. The quantity and distribution of polymorphic bases observed in this group (specified below) meant that there was a choice of regions that could be used for differentiation. Following testing of four possible regions, the region encompassing amino acids 248–250 was selected for use in the final non-g assay. Each serotype had a unique motif at the target region except *fliC-l,v *and *fliC-l,z*_13 _which shared a sequence type (Figure 4).

Some amino acid sequences were not identical within non-g alleles, including i, r, d, e,h, a and z_4_,z_23 _(Additional file 1). A previous study of *fliC-i *sequences reported no variation in a 260 bp region among seven Typhimurium strains [17]. Six full *S*. Typhimurium *fliC*s and a fragment spanning nucleotides 434–1090, corresponding to amino acids 159 – 400, of a further 20 *S*. Typhimurium strains were sequenced. Three distinct DNA sequences which resulted in translated differences in the expressed peptides were observed within the serotype. Sequence type "Typhimurium_a" was detected in 18 strains, identical to the sequenced strain LT2. Sequence type "Typhimurium_b" was detected in four strains and was differentiated by a SNP at 768, conferring a 256 Glu>Lys substitution. Sequence type "Typhimurium_c" conferred a Glu>Lys substitution and an Ala>Thr substitution at 263 and was found in two strains: 571896 and 571913. Strains 571896 and 571913 were phage type DT104 however, other strains tested did not conform to recognised phage typing patterns so no assured correlation could be made with phage type or other phenotype. *S*. Choleraesuis sequence (*fliC-c*) differed from that published (AF159459) at one nucleotide, conferring amino acid substitution of Thr >Ser at codon 99.

*FliC *sequences of nine *S*. Heidelberg strains were identical, consistent with the results of a previous report [18]. The published sequence for *fliC-r *of *S*. Rubislaw (X04505) differed from *S*. Heidelberg at three amino acids. The *S*. Muenchen sequence determined in this study differs in twelve amino acids to the published *S*. Muenchen (X03395), and differed in 25 amino acids from the *S*. Duisberg sequence in this study. *S*. Anatum, *S*. Newport and *S*. Saintpaul exhibit factors e,h in their phase 1 flagellar. Amino acid sequence was conserved in two strains of *S*. Saintpaul but distinct for each serotype due to four amino acid substitutions at codons 192, 213, 238, 356. *S*. Brandenburg and *S*. Panama exhibit l,v in the phase 1 antigen, no inferred amino acid differences were detected. *FliC-l,v *sequences clustered with *fliC-l,z*_13_(Figure 1).

*FliC *from three strains exhibiting the z_4 _antigenic factor in phase 1 flagellar were sequenced. Cluster analysis grouped these sequences together in the non-g group although they contain regions of sequence similar to g-complex strains (amino acid positions 96 – 164). Z_4_,z_24 _is distinct from z_4_,z_23 _and z_4_,z_23 _sequences varied within the serotype at seven amino acid positions: 235, 237, 239, 242, 253, 351 and 369. The complex mosaic nature of *fliC *is evident from analysis of amino acid alignment of sequences in particular strains from subgroups in the SARC collection (see Materials and Methods).

### Molecular serotyping assays

By comparison of amino acid sequences coding for antigens of the different serotypes, sero-specific motifs were identified. Individual regions of *fliC *were selected for the g-group and non-g group to provide unique sequence for as many serotypes as possible, while keeping the assay simple to perform and analyse. Two multiplex PCRs were developed for the production of *fliC *amplicon of g-complex strains and *fliC *amplicon of non-g strains. Sero-specific motifs in each amplicon were consequently identified by sequencing-by-synthesis.

### G-complex assay

Fifteen sequence types were identified in the 17 bp of nucleotide sequence assayed (Figure 3). Twenty-seven strains were tested and each produced a recognised sequence motif which differentiated between serotypes. Serotypes would be fully resolved through the detection of further polymorphisms, for example g, [s],t and g,t can be separated through additional detection of a A>G change at nucleotide position 777 conferring amino acid Ser>Gly substitution specific to g,t.

### Non-g assay

Fourteen sequence types were identified in the 9 bp of nucleotide sequence assayed (Figure 4). Thirty strains were tested, each producing a recognised sequence motif allowing separation of serotypes. Serotypes l,v and l,z_13 _gave the same motif at the target region but could be separated by nucleotide substitution A>G at position 783 conferring a Thr>Ala change.

The stability of the targeted polymorphisms in *Salmonella *phase 1 flagellar antigens was demonstrated through testing on a panel of 55 isolates. The SNP responsible for the antigenic difference between serotypes g,m and g,p was within the target region and so could be differentiated by the assay. The amino acid substitution that separated *fliC-g,p,u *was also encoded within the sequence assayed. Antigens i, r, c, d, b, e,h, k, a, z_41_, z, z_10_, z_4_,z_23_, z_4_z_24_, g,q, g,m,p, g,p,u, [f],g,t, g,z_51 _and biotype *S*. Pullorum gave unique motifs, l,v and l,z_13 _shared a motif. Some serotypes for which certain factors may be present or absent (denoted by square brackets in antigenic formulae) were not separated from similar serotypes: [f],g,m, [p], g,m and g,m, [p],s; [f],g,m, [p] and g,m, [t]; g, [s],t and g,t although these could be separated by other DNA polymorphisms as discussed. Two motifs were observed for k, each specific to *S*. Thompson and IIIb. Two motifs were observed for d, specific to *S*. Duisberg and *S*. Muenchen / *S*. Schwarzengrund. Published sequence data for *fliC-m,t*, from serotypes *S*. Banana, *S*. Oranienburg and *S*. Pensacola were included in assay design. The polymorphic region targeted by the assay is predicted to differentiate m,t sequences from other g-complex antigens, and also differentiate *S*. Pensacola from *S*. Banana and *S*. Oranienberg. Strains exhibiting factors m,t were not available for testing.

## Conclusions

A high level of sequence homology between *fliC *genes of g-complex strains was observed. Data produced for this study is congruent with a previous report of g-complex sequences [16]. The genetic basis between distinct antigens in this group of sequences can be a single amino acid substitution. Specific motifs could be identified as the genetic basis for particular antigenic differences and hence their involvement in epitope formation and stability among strains inferred.

Full gene sequences were distinct for each antigen analysed in this study. Furthermore, analysis of multiple representatives revealed that some antigens were encoded for by multiple sequences. In these cases DNA sequence based methods are more discriminatory than traditional serotyping methods which do not recognise these as distinct antigens.

Assays were designed such that an unknown strain could be identified in respect of its phase 1 flagellar antigen in two steps. The specific PCR acted as the first level of identification and the resultant amplicon was used for the pyrosequencing assay. A positive PCR indicated which of two Pyrosequencing assays to apply. Each assay was uniform in that only one mix of pyrosequencing primers and one dispensation order was needed. All the strains tested were successfully amplified by PCR. As some analyses have been performed on unpublished data, exhaustive testing of the assay will be performed to confirm specificity and typeability of all recognised serotypes.

Molecular serotyping will incorporate the desirable properties of serology (typeability, reproducibility, epidemiological significance) together with the advantages of DNA analysis (ability to automate, labour saving, serum independent). Antisera production and associated quality control measures would be unnecessary for a DNA sequence based method. Time-consuming flagellar phase reversal to identify both flagellar antigens is not necessary at the genetic level. Other advantages include reduced labour costs, rapid results in comparison to traditional serotyping methods. DNA sequence data is highly portable and easy to interpret. The method described was easily automated by use of the vacuum preparation tool for the DNA strand separation step and could be further automated by use of robotics for PCR set-up. Result output included a pyrogram, raw text and confidence level; automation of data analysis could be achieved by use of a computer script to screen at a set confidence level and cross-check results against a database of recognised motifs.

With the capability to identify approximately 97% of phase 1 flagellar antigens from medically important *Salmonella *strains occurring in England and Wales, the assay can be used now as an economic screen of unknown isolates and alleviate the burden on routine serotyping work. A scheme including the phase 1 flagellar assay and complementary assays for phase 2 flagellar and polysaccharide antigens is currently being piloted and based on incidence data of the top 50 serotypes from 2003, it is anticipated that the scheme will provide a complete molecular serotype for around 80% of isolates and confident prediction of 76% of the remainder.

### Future work

Alternative sero-specific polymorphisms identified in this study could be exploited by similar assays to allow further separation when antigens did not give unique pyrograms.

The alliance of the *fliC *assay to a *fljB *and *rfb *assay would allow the full antigenic formulae of *Salmonella *serotypes to be determined. Common phase 2 flagellar antigens will be selected for sequencing and together with published data will be analysed for sero-specific motifs and a short sequence assay designed with the approach described in this study.

In 1993, Luk *et a*l [8] outlined a simple length heterogeneity PCR for identification of *Salmonella *major serogroups A, B, C2, and D. They based their PCR on the presence/absence of genes or sequence polymorphisms within shared genes. Essentially, only serogroups A and D possess a gene to synthesise tyvelose but serogroup A genes carry an early stop codon and do not produce the sugar itself. Only groups B and C2 possess a gene to synthesise abequose but the sequences are distinct. We have also designed a preliminary pyrosequencing assay to distinguish these serogroups based on amplification and short sequences of these genes (data not shown).

In summary, epitopes are conformational and it is difficult to determine which amino acids would interact from a linear sequence. However, in the g-complex sequences some amino acid changes could be identified as responsible for differences in antigenic factors because variation was minimal. There is no common factor among the non-g antigens and the sequences are much more heterogenous; there are too many substitutions to draw conclusions about epitope specific sequences. Epitope mapping could be used to further investigate epitopes responsible for antigenic specificity.

## Methods

### Bacterial strains

Strains exhibiting the different phase 1 flagellar antigenic factors were selected from *Salmonella *Reference Collections A, B and C obtained from the University of Calgary. Multiple isolates of *S*. Enteritidis phage type 4, and *S*. Typhimurium phage type DT104 plus a panel of serotyped strains were gratefully received from the *Salmonella *Reference Laboratory, Health Protection Agency, Colindale (Additional file 2).

### DNA preparation, PCR and sequencing

MagNA Pure instrument and Total Nucleic Acid Extraction Kit 1 (Roche, East Sussex). PCR reactions contained 1X PCR buffer, 20 pmoles of FL_START2, 20 pmoles rFSa1 [21], 1 U Taq polymerase, 0.25 mM of each dNTP, 4 mM MgCl_2 _(Sigma-Aldrich, Dorset).

PCR amplification of the *fliC *gene was performed with an 9700 GeneAmp PCR System (Applied Biosystems, Cheshire): 35 cycles of 95°C for 60 sec, 50°C for 60 sec, 72°C for 30 sec followed by a 7 min final extension at 72°C. PCR products were purified with Qiaquick spin columns (Qiagen Ltd, West Sussex) and quantitated by gel electrophoresis using Ready-to-Run pre-cast gels (Amersham Biosciences, Buckinghamshire). Fifty to one-hundred nanograms of the purified PCR product was used for cycle sequencing, with specific primers (Additional file 3) and the CEQ DTCS dye terminator kit (Beckman Coulter, Buckinghamshire). Excess dNTPs were removed from sequencing reactions using GenClean, a 96-well plate format gel filtration system (Genetix Ltd, Hampshire). Sequencing reactions were run on a CEQ 8000XL capillary sequencer (Beckman Coulter). Primers were designed on generated sequence aided by Eprimer3 [22] in a primer walking approach to complete sequencing of the full gene. Sequences generated have been submitted to GenBank (Accession numbers AY649696-AY6497242).

### Sequence analysis

Data were analysed and assembled using SeqMan, a component of the DNA Star software package. Multiple alignments were created using BioEdit (Tom Hall, North Carolina State University).

Phylogeny inference package Phylip (Joe Felsenstein, University of Washington) was used to compute a distance matrix from protein sequences and build trees illustrating the relatedness of *fliC *sequences. Some previously published sequences were included (Additional file 3). Polymorphisms postulated to be serotype specific were identified from the alignments of full *fliC *sequences; in-house programme MOP-UPs [23] identified motifs and designed primers to user-specified groups of sequences in the alignment (Anthony Underwood, Health Protection Agency, London).

### Assay design

Two multiplex PCRs were designed to amplify polymorphic regions of both g- and non-g complex *fliC *sequences. Amplicon sizes were approximately 316 bp for g-complex strains, 170 bp – 250 bp, (size varied according to serotype) from non-g strains. The order of nucleotide dispensation was tailored to enable the first two dispensations to act as negative and positive controls.

### DNA preparation and Pyrosequencing

A 1 μl loop of cells was boiled in 100 μl of sterile distilled water for 10 min at 95°C. One microlitre of lysate was used for PCR. Two PCRs were run in parallel to amplify fragments of the *fliC *gene. Fifty microlitres of PCR product was prepared containing 1 U Taq polymerase, 0.25 mM of each dNTP, 4 mM MgCl_2_. PCR for amplification of g-complex strains used three forward primers: 100 pmol GPYRO-A; 12.5 pmol GPYRO-B; and 12.5 pmol GPYRO-C; and 125 picomoles of reverse biotinylated primer G-REV. PCR for amplification of non-g strains used 14 forward primers (NON-G-PYRO-A, NON-G-PYRO-B etc.) in equal concentrations. Thirteen biotinylated reverse primers (NON-G-REV-A, NON-G-REV-B etc.) were used in equal concentrations. In each 50 μl reaction 125 pmol of mixed forward primer and 125 pmol mixed reverse primer was used. Primer sequences are detailed in Additional file 3.

Thermocycling was performed with an Applied Biosystems 9700 GeneAmp PCR System using a touch-down programme: initial denaturation step of 94°C for 2 min; followed by 17 cycles of 94°C for 20 sec, 66°C (-1°C per cycle) for 30 sec, 72°C for 30 sec; followed by 20 cycles of 94°C for 20 sec, 54°C for 30 sec, 72°C for 30 sec. The excess primers were removed using a filter plate and vacuum system (Genetix Ltd, Hampshire) before visualising the PCR products on the Ready-To-Run agarose system (as previously). Biotinylated single-stranded DNA was immobilized on streptavidin-coated sepharose beads (Amersham Biosciences, Buckinghamshire) with binding buffer. The mixture was agitated at 1400 rpm for 10 min at room temperature. Single stranded DNA bound to beads was isolated from the mixture using a series of wash steps for 5 seconds each in turn, 70% ethanol, 0.2M NaOH and washing buffer. Ninety-six samples were prepared in 2 minutes by automation of strand separation step using a vacuum preparation tool (Pyrosequencing AB, Uppsala, Sweden). A combination of pyrosequencing primers was used for each assay; 20 primers for the non-g complex assay, and three for the g-complex assay (Additional file 3) into which DNA was eluted. Pyrosequencing primers were annealed to single-stranded DNA on the beads by heating to 80°C for 2 minutes and allowed to cool slowly. Single stranded binding protein, enzyme mix, substrate mix and dNTPs (Pyrosequencing) were added sequentially by the instrument according to the programmed dispensation order.

## Authors' contributions

CM carried out the sequencing, constructed the multiple alignments and designed the assays. TP carried out the serotyping. TP advised on *Salmonella *serotyping and provided strains. CA CM SG TP and JL participated in the design of the study. CA conceived of and coordinated the study. All authors read and approved the final manuscript.

## Supplementary Material

Additional File 1 Amino acid alignment. Amino acid alignment of 106 *fliC *gene sequences representing 32 H1 alleles. Sequences labelled with _a, _b or _c indicate an H1 allele encoded by multiple sequences (Additional file 2). Codon numbering is in reference to the sequence of Typhimurium_a which represents sequenced strain LT2. Click here for file

Additional File 3 Primers used for PCR and Pyrosequencing. Orientation of the primer is represented by F (forward) or R (reverse) and approximate position is given as nucleotide distance from 5' end of fliC *These primers were also used as pyrosequencing primers Click here for file

Additional File 2 Sequences used in this study. Sequences labelled with _a, _b or _c indicate an H1 allele encoded by multiple sequences Click here for file
