# Supplementary material for: Towards the development of a DNA-sequence based approach to serotyping of Salmonella enterica
Source: BMC Microbiol. 2004 Aug 6;4:31. doi: 10.1186/1471-2180-4-31 (PMC514894; doi:10.1186/1471-2180-4-31)
Supplement: Additional File 1 — Amino acid alignment. Amino acid alignment of 106 fliC gene sequences representing 32 H1 alleles. Sequences labelled with _a, _b or _c indicate an H1 allele encoded by multiple sequences (Additional file 2). Codon numbering is in reference to the sequence of Typhimurium_a which represents sequenced strain LT2. [file 1471-2180-4-31-S1.pdf]

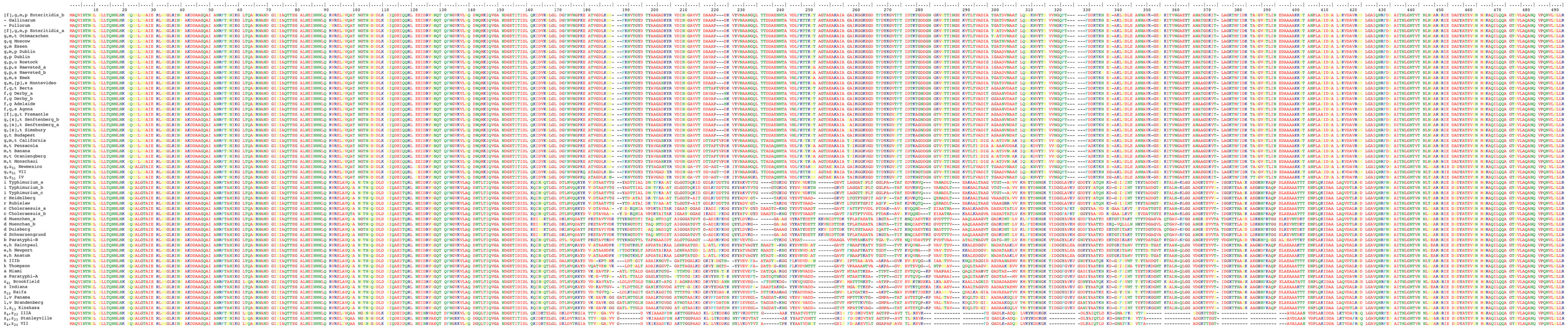

[f],g,m,p Enteritidis_b
- Gallinarum
- Pullorum
[f],g,m,p Enteritidis_a
g,m,t Othmarschen
g,q Moscow
g,m Essen
g,m,p Dublin
g,p Dublin
g,p,u Rostock
g,p,s Naevsted_a
g,p,s Naevsted_b
g,m,s Emek
g,m,[p],s Montevideo
f,g,t Berta
f,g Derby_a
f,g Derby_b
f,g Adelaide
f,g,s Agona
[f],g,t Fremantle
g,[s],t Senftenberg_b
g,[s],t Senftenberg_a
g,[s],t Simsbury
g,t Budapest
g,m,t California
m,t Pensacola
m,t Banana
m,t Oraniengberg
m,t Monschaui
g,z_{51} Newmexico
g,z_{51} VII
g,z_{51} IV
i Typhimurium_a
i Typhimurium_b
i Typhimurium_c
r Heidelberg
r Rubislaw
c Choleraesuis_a
c Choleraesuis_b
d Muenchen_a
d Muenchan_b
d Duisberg
d Schwarzengrund
b Paratyphi-B
e,h Saintpaul
e,h Newport
e,h Anatum
k IIIb
k Thompson
a Miami
a Paratyphi-A
z_{41} Brookfield
z Indiana
z_{10} Haifa
l,v Panama
l,v Brandenberg
l,z_{13} Kinshasa
z_4,z_{23} IIIA
z_4,z_{23} Stanleyville
z_4,z_{24} VII
