# Supplementary material for: Towards the development of a DNA-sequence based approach to serotyping of Salmonella enterica
Source: BMC Microbiol. 2004 Aug 6;4:31. doi: 10.1186/1471-2180-4-31 (PMC514894; doi:10.1186/1471-2180-4-31)
Supplement: Additional File 3 — Primers used for PCR and Pyrosequencing. Orientation of the primer is represented by F (forward) or R (reverse) and approximate position is given as nucleotide distance from 5' end of fliC *These primers were also used as pyrosequencing primers [file 1471-2180-4-31-S3.pdf]

	PRIMER NAME	LABEL	PRIMER SEQUENCE
***fliC* PCR PRIMERS**			
	FL_START2		ATGGCACAAGTCATTAATAC
	rFSa1		TTAACGCAGTAAAGAGAGGAC
**G-COMPLEX PCR PRIMERS**			
**FORWARD**			
	G-PYRO-A*		CGTTAACTGTCGCTGATAT
	G-PYRO-B*		CATTAACAATTAGTGATAT
	G-PYRO-C*		CGTTGACTGTTGCTGAYAT
**REVERSE**			
	G-REV	5’ BIOTIN	ACTKRCCRYTYACWACRGA

NON G-COMPLEX PCR PRIMERS

**FORWARD**			
	NON-G-PYRO-A*		TAAARATGGTGTTTATGA
	NON-G-PYRO-B*		GTAAGAATGGTAAATATGA
	NON-G-PYRO-C*		TAAAAATGGAACCTATG
	NON-G-PYRO-D*		TAAAAATGGTTTCCTTAA
	NON-G-PYRO-E*		TAAAAATGGCTACTATAA
	NON-G-PYRO-F*		CCAAAAATGGYRAWTATGA
	NON-G-PYRO-G*		GAAAGATGGTTTCTACAA
	NON-G-PYRO-H*		CAAAGGCGGATTCTATAA
	NON-G-PYRO-I*		GCATGCTGGTTTCTACAA
	NON-G-PYRO-J*		TAAAGGCGGTTTTCTTAA
	NON-G-PYRO-K*		TGAAGCGACAGTTGATGC
	NON-G-PYRO-L*		TAAAGCMACTTATGATGA
	NON-G-PYRO-M*		ACTAAAGGAATCGCAACCACTGTA
	NON-G-PYRO-N*		TAAAGATGGCTATTATGA
**REVERSE**			
	NON-G-REV-A	5’ BIOTIN	AAACGCCACCATCTTCTA
	NON-G-REV-B	5’ BIOTIN	TGCGAACCCACCATCAATCGTC
	NON-G-REV-C	5’ BIOTIN	ACAGCATAACCACCATCAATCGTC
	NON-G-REV-D	5’ BIOTIN	GCAACCGCATCAGCAATACCAC
	NON-G-REV-E	5’ BIOTIN	ACAGCATAACCGCCATCAATCGTC
	NON-G-REV-F	5’ BIOTIN	TTAACACCGAAGCCGCCATCAATCGTC
	NON-G-REV-G	5’ BIOTIN	AAACGCCCCCATCTTGTA
	NON-G-REV-H	5’ BIOTIN	ATACGCCCCCATCTTGTA
	NON-G-REV-I	5’ BIOTIN	ACACCGAAACCGCCATCAATAGTC
	NON-G-REV-J	5’ BIOTIN	TGGCGTCGGTGTAACAGCGTCTA
	NON-G-REV-K	5’ BIOTIN	CACCTGCTGCTGTCAATG
	NON-G-REV-L	5’ BIOTIN	ACTGCATAGCCACCATCAATAACC
	NON-G-REV-M	5’ BIOTIN	ATCGGCTTTTARATCTGCTCCAT

***fliC* SEQUENCING**

POSITION AND ORIENTATION	PRIMERID	PRIMER SEQUENCE
F0001	PRIMER-2	CAAGTCATTAATACAAACAGCC
F0285	PRIMER-3	TTCAGGCCACTAACGGGACTAA
F0380	PRIMER-12	ACCCAGTTCAACGGCGTGAAAG
F0390	PRIMER-16	ACTCAGTTCAACGGCGTGAAAG
F0415	PRIMER-4	TCTCAGGACAACCAGATGAAAATC
F0427	PRIMER-11	AACGATGGCGAGACTATTACC
F0427	PRIMER-113	GTTACGTTGACTGTCGCTGAC
F0456	PRIMER-13	AAAGTCTTGGTCTGGATGG
F0480	PRIMER-41	GAAGCAGATCAACTCTCAGACC
F0507	PRIMER-10	CTCTGGAGCTTCGGTAGTTG
F0516	PRIMER-8	AGCCTCGGTAGTTGGTGAT
F0556	PRIMER-14	GTCCCGCAGCAGATAAATTA
F0570	PRIMER-5	CAAGAATGTTACGGGTTACGAC
F0649	PRIMER-7	AGGCGATTCCTTGTCTGCTA
F0720	PRIMER-23	AATAACACTGCGGTTGACCTC
F0730	PRIMER-15	CGGTTGATCTCTTTAAGACCAC
F0805	PRIMER-28	AGGACCAATTACTGCTGGCTTC
F0810	PRIMER-124	CCCCGCTTACAGGTGGACTAC
F0850	PRIMER-27	CCAAAGTACAGGTTAATGCTCC
F0852	PRIMER-6	ACTGTGACCGGCGCTGGATACT
F0875	PRIMER-26	AGAAAGCAATTAAGGCGTCTGC
F0915	PRIMER-20	GAAGATGGTGGCGTTTCAAATG
F0915	PRIMER-25	AAAGCGAGTGGTATTGCTGATG
F0930	PRIMER-30	CTGGTGCCGATAAGGACAATAC
F0940	PRIMER-33	AGACTTGGCTGCAAACGT
F0940	PRIMER-37	AGCCTTGGCTGCAAACGT
F0975	PRIMER-36	ATGCMGCTACCTTACAATCAAG
F0975	PRIMER-17	ATGCTGCTACCTTACAATCAAG
F0988	PRIMER-9	ATTAACGCCACTGAATATACCG
F0990	PRIMER-38	AACACTACGTCTTACACTGCAGAT
F0990	PRIMER-119	ATCTATTGACGGTGGTTATGCG
F0990	PRIMER-29	CAAAGTTACTGATGCGGGTTAC
F0995	PRIMER-24	GGTTATTGATGGTGGCTATGCA
F1000	PRIMER-118	GATGGTGGTTTAGCAGTTAAGG
F1010	PRIMER-21	TAAGACTATTGATGGCGGTTTC
F1027	PRIMER-40	GAATCTGCCATCACTAACCTG
F1040	PRIMER-22	CAGCATCTGTTGATAAATCTGG
F1054	PRIMER-31	ACTGCGGCTAACCAACTT
F1092	PRIMER-18	GGCGCAGACGGCAAAACC
F1095	PRIMER-19	ACAATGCTGTTAAAGGCGAAAG
F1098	PRIMER-39	TAGACGGCAAAACCGAAGTT
F1130	PRIMER-217	CCGTGCTGCGAAAATGTCC
F1144	PRIMER-42	AGATCACCTTAGCTGGCA
F1154	PRIMER-32	AGGTCACCTTAGCTGGCA
R0390	PRIMER-85	TCACGCCGTTGAACTGAG
R0400	PRIMER-103	TGCGCCAGGACTTTCACG
R0410	PRIMER-101	TTTCATCTGGTTGTCCTGAGAC
R0415	PRIMER-75	GATTTTCATCTGGTTGTCCTGAG
R0416	PRIMER-112	AAGATCGATTGTGATAGTTTCAC
R0416	PRIMER-110	AAGATCGATTGTGATAGTTTCGC
R0445	PRIMER-107	TTTCACCGTCGTTGGCAC
R0450	PRIMER-105	ATCAATAGTTTCACCGTCGTTC
R0463	PRIMER-109	GCACATTCAGCGTATCCAGA
R0480	PRIMER-104	GGTCTGAGAGTTGATCTGCTTC
R0484	PRIMER-115	TTGCTTGTTTAGGCCCATTC
R0508	PRIMER-116	AATCAGCTTCTTTGATCTTAACAT
R0566	PRIMER-114	ATCAGCACCAACAGCATACG
R0570	PRIMER-117	GTCGTAACCCGTAACATTCTTG
R0635	PRIMER-96	CTTAATTGCAGCAGTACCGTTTGT
R0637	PRIMER-87	CGTCGCAGTAACATCTTTG
R0700	PRIMER-215	AGCCGCGTTAACAAAGACAGC
R0743	PRIMER-89	TAGTTACTGCTGGCGGAG
R0755	PRIMER-108	TGTCGCTTCATACAGACCATC
R0760	PRIMER-69	TCGCACTATCAACATTCACTTC
R0800	PRIMER-106	TCCTTCCTTACCACCTTTAATG
R0832	PRIMER-90	AGCATCAATACCGCCTGC
R0865	PRIMER-71	CCGTCATCACCAGTTTTTGTATCA
R0905	PRIMER-88	CCGCCATCAATCGTCTTAC
R0915	PRIMER-92	AGAGCTGCTTTAACCTCTGCA
R0920	PRIMER-78	CATTTGAAACGCCACCATC
R0927	PRIMER-94	ATTTGAAAGTTGCATTCCCG
R0950	PRIMER-91	GTCAGCGACAGTCAACGTAAC
R0960	PRIMER-82	TTTCATCGTATCAGCGGC
R0970	PRIMER-68	TAAGGTAGCAGCATCAACATTC
R0975	PRIMER-93	CTTGATTGTAAGGTAGCAGCAT
R0990	PRIMER-77	CATAACCACCATCAATCGTCT
R0990	PRIMER-73	AGCCACCATCAATAACCTTACC
R0995	PRIMER-76	CCGTATCCACCATCAATAGTT
R0995	PRIMER-98	CGCATAACCACCGTCAATAGAT
R1000	PRIMER-86	CCACCATCTTCTAGCGCTTT
R1000	PRIMER-214	GTTTTAGTGTTGCCGCCTTTATCG
R1000	PRIMER-81	CCTTAACTGCTAAACCACCATC
R1004	PRIMER-111	AGTTTCCGCACCTCTCATT
R1025	PRIMER-72	ATGTAGCGGCATAGAAATCGTC
R1025	PRIMER-83	GTAGCGGCATAGAAATCGTC
R1030	PRIMER-100	ATGCGAACCCACCATCAATAAC
R1036	PRIMER-67	ACCGTCATCTGCAGTGTATTTCGTAG
R1060	PRIMER-74	CAGAAAGTTTCGCACTCTCG
R1065	PRIMER-99	AGTGGTGTTAATGCTGAACGATC
R1090	PRIMER-80	CCCGTGGAATCAGTATAAGTGG
R1098	PRIMER-84	GGCTTTGCTGGCATTGTAGGTTTT
R1130	PRIMER-216	TTTTCGCAGCACGGTTATCG
R1135	PRIMER-97	GTGGCGTTAGCAGTATATTCAG
R1155	PRIMER-70	TAAGGTGACCTTATCACCCGTG
R1495	PRIMER-64	TTTGCGGAACCTGGTTAG
R1515	PRIMER-63	TTAACGCAGTAAAGAGAGGAC
R1515	PRIMER-65	ACGCAGTAAAGAGAGGACGTT
