# Supplementary material for: Towards the development of a DNA-sequence based approach to serotyping of Salmonella enterica
Source: BMC Microbiol. 2004 Aug 6;4:31. doi: 10.1186/1471-2180-4-31 (PMC514894; doi:10.1186/1471-2180-4-31)
Supplement: Additional File 2 — Sequences used in this study. Sequences labelled with _a, _b or _c indicate an H1 allele encoded by multiple sequences [file 1471-2180-4-31-S2.pdf]

H1 antigen	Serotype	Strain identifier	Sequence identifier	Accession number	Reference
-	Gallinarum	M84975	Gallinarum	M84975	Li 1993
-	Gallinarum	M84976	Gallinarum	M84976	Li 1993
-	Gallinarum	M84979	Gallinarum	M84979	Li 1993
-	Pullorum	B51	Pullorum	AY649696	This study
-	Pullorum	M84977	Pullorum	M84977	Li 1993
a	Miami	B28	Miami	AY649697	This study
b	Paratyphi-B	A41	Paratyphi-B	AY649698	This study
c	Choleraesuis	AF159459	Choleraesuis_b	AF159459	Unpublished
c	Choleraesuis	B04	Choleraesuis_a	AY649740	This study
d	Duisberg	B15	Duisberg	AY649700°	This study
d	Muenchen	A63	Muenchen_a	AY649701	This study
d	Schwarzengrund	B57	Schwarzengrund	AY649732	This study
e,h	Anatum	B02	Anatum	AY649702	This study
e,h	Newport	B36	Newport	AY649703	This study
e,h	Saintpaul	A22	Saintpaul	AY649704	This study
f,g	Adelaide	U05295	Adelaide	U05295	Masten
f,g	Derby	B09	Derby_a	AY649707	This study
f,g	Derby	U06225	Derby_b	U06225	Li 1994
f,g,s	Agona	B01	Agona	AY649705	This study
f,g,t	Berta	U06227	Berta	U06227	Li 1994
[f],g,t	Fremantle	C04	Fremantle	AY649706	This study
[f],g,t	Fremantle	U06197	Fremantle	U06197	Li 1994
[f],g,m,[p]	Enteritidis	B16	Enteritidis_b		This study
[f],g,m,[p]	Enteritidis	B17	Enteritidis_a		This study
[f],g,m,[p]	Enteritidis	B18	Enteritidis_b	AY649709	This study
[f],g,m,[p]	Enteritidis	JTCM01	Enteritidis_b	AY649741	This study
[f],g,m,[p]	Enteritidis	M84980	Enteritidis_a	M84980	Selander 1992
[f],g,m,[p]	Enteritidis	M84974	Enteritidis_b	M84974	Selander 1992
[f],g,m,[p]	Enteritidis	571747	Enteritidis_b		This study
[f],g,m,[p]	Enteritidis	571767	Enteritidis_b		This study
[f],g,m,[p]	Enteritidis	571773	Enteritidis_b		This study
[f],g,m,[p]	Enteritidis	571786	Enteritidis_b		This study
[f],g,m,[p]	Enteritidis	571790	Enteritidis_b		This study
[f],g,m,[p]	Enteritidis	571812	Enteritidis_b		This study
[f],g,m,[p]	Enteritidis	571862	Enteritidis_b		This study
[f],g,m,[p]	Enteritidis	571871	Enteritidis_b		This study
[f],g,m,[p]	Enteritidis	571885	Enteritidis_b		This study
[f],g,m,[p]	Enteritidis	571888	Enteritidis_b		This study
[f],g,m,[p]	Enteritidis	571909	Enteritidis_b		This study
[f],g,m,[p]	Enteritidis	571910	Enteritidis_b		This study
[f],g,m,[p]	Enteritidis	571924	Enteritidis_b		This study
[f],g,m,[p]	Enteritidis	571926	Enteritidis_b		This study
[f],g,m,[p]	Enteritidis	571929	Enteritidis_b		This study
[f],g,m,[p]	Enteritidis	571930	Enteritidis_b		This study
[f],g,m,[p]	Enteritidis	571931	Enteritidis_b		This study
[f],g,m,[p]	Enteritidis	571933	Enteritidis_b		This study
[f],g,m,[p]	Enteritidis	571935	Enteritidis_b		This study
[f],g,m,[p]	Enteritidis	571946	Enteritidis_b		This study
g,[s],t	Simsbury	U05303	Simsbury	U05303	Unpublished
g,[s],t	Senftenberg	B59	Senftenberg_a	AY649714	This study
g,[s],t	Senftenberg	Z15072	Senftenberg_b	Z15072	Masten 1993
g,m	Essen	U05299	Essen	U05299	Unpublished
g,m,[p],s	Montevideo	B31	Montevideo	AY649708	This study
g,m,[p],s	Montevideo	Z15069	Montevideo	Z15069	Masten 1993
g,m,[t]	Othmarschen	U06455	Othmarschen	U06455	Li 1994
g,m,s	Emek	JTCMO2	Emek	AY649742	This study
g,m,t	California	U05296	California	U05296	Unpublished
g,m,p	Dublin	M84972	Dublin_b	M84972	Selander 1992
g,p	Dublin	B12	Dublin_a	AY649712	This study
g,p	Dublin	M84973	Dublin_a	M84973	Selander 1992
g,p	Dublin	Z15067	Dublin_a	Z15067	Masten 1993
g,p,s	Naevsted	D78639	Naevsted_b	D78639	Unpublished
g,p,s	Naevsted	JT473	Naevsted_a	AY649710	This study
g,p,u	Rostock	JT66	Rostock	AY649711	This study
g,p,u	Rostock	Z15071	Rostock	Z15071	Masten 1993
g,q	Moscow	JT67	Moscow	AY649713	This study
g,q	Moscow	Z15086	Moscow	Z15086	Masten 1993
g,t	Budapest	JT26	Budapest	AY649715	This study
g,t	Budapest	Z15065	Budapest	Z15065	Masten 1993
g,z_{51}	IV	C09	IV	AY649716	This study
g,z_{51}	VII	C15	VII	AY649717	This study
g,z_{51}	Newmexico	U06199	Newmexico	U06199	Li 1994
i	Typhiumrium	571913	Typhiumrium_a	AY649733	This study
i	Typhiumrium	571896	Typhiumrium_a		This study
i	Typhiumrium	A01	Typhiumrium_b		This study
i	Typhiumrium	A13	Typhiumrium_c	AY649718	This study
i	Typhiumrium	A16	Typhiumrium_b	AY649719	This study
i	Typhiumrium	A18	Typhiumrium_c		This study
i	Typhiumrium	A19	Typhiumrium_c	AY649720	This study
i	Typhiumrium	C01	Typhiumrium_b	AY649721	This study
i	Typhiumrium	AE008787	Typhiumrium_b	AE008787	McClelland 2001
k	Thompson	B62	Thompson	AY649722	This study
l,v	Brandenburg	B03	Brandenburg	AY649723	This study
l,v	Panama	B39	Panama	AY649724	This study
l,z_{13}	Kinshasa	JT256	Kinshasa	AY649725	This study
m,t	Banana	U06202	Banana	U06202	Li 1994
m,t	Monschaui	U05302	Monschaui	U05302	Unpublished
m,t	Oranienburg	U06201	Oranienburg	U06201	Li 1994
m,t	Pensacola	U06200	Pensacola	U06200	Li 1994
r	Heidelberg	A30	Heidelberg	AY649727	This study
r	Heidelberg	A31	Heidelberg	AY649728	This study
r	Heidelberg	A32	Heidelberg	AY649729	This study
r	Heidelberg	A35	Heidelberg	AY649730	This study
r	Heidelberg	A36	Heidelberg	AY649731	This study
r	Heidelberg	A37	Heidelberg		This study
r	Heidelberg	A40	Heidelberg		This study
r	Heidelberg	B23	Heidelberg	AY649726	This study
r	Rubislaw	X04505	Rubislaw	X04505	Wei 1986
z	Indiana	B25	Indiana	AY649739	This study
z_{10}	Haifa	B22	Haifa	AY649734	This study
z_4,z_{23}	Stanleyville	B61	Stanleyville	AY649736	This study
z_4,z_{23}	IIIa	C05	z_4,z_{23}_IIIa	AY649735	This study
z_4,z_{24}	VII	C16	z_4,z_{24}_VII	AY649737	This study
z_{41}	V	C11	z_{41}_V	AY649738	This study
